# Transcriptional Profiling Analysis Providing Insights into the Harsh Environments Tolerance Mechanisms of *Krascheninnikovia arborescens*

**DOI:** 10.3390/ijms252211891

**Published:** 2024-11-05

**Authors:** Hongyi Zhang, Yingnan Wang, Binjie Ma, Xiangqi Bu, Zhenhua Dang, Yingchun Wang

**Affiliations:** 1 Ministry of Education Key Laboratory of Forage and Endemic Crop Biology, School of Life Sciences, Inner Mongolia University, Hohhot 010070, China; zhanghy623@163.com (H.Z.); 15765369556@163.com (Y.W.); buxq1998@163.com (X.B.); 2 Institute of Crop Sciences (ICS), Chinese Academy of Agricultural Sciences (CAAS), Beijing 100081, China; bjma055@163.com; 3 Hainan Yazhou Bay Seed Laboratory, Sanya 572025, China; 4 National Nanfan Research Institute (Sanya), Chinese Academy of Agricultural Sciences, Sanya 572024, China; 5 Ministry of Education Key Laboratory of Ecology and Resource Use of the Mongolian Plateau & Inner Mongolia Key Laboratory of Grassland Ecology, School of Ecology and Environment, Inner Mongolia University, Hohhot 010070, China

**Keywords:** *Krascheninnikovia arborescens*, RNA-Seq, abiotic stress, plant hormones, phenylpropane metabolism, transcription factor, WGCNA

## Abstract

*Krascheninnikovia arborescens*, an endemic shrub in China, thrives in desertification-prone environments due to its robust biomass, hairy leaves, and extensive root system. It is vital for ecological restoration and serves as a valuable forage plant. This study explored the molecular mechanisms underlying *K. arborescens*’ adaptation to desert conditions, focusing on its physiological, biochemical, and transcriptomic responses to drought, salt, and alkali stresses. The results revealed that the three stresses have significant impacts on the photosynthetic, antioxidant, and ion balance systems of the plants, with the alkali stress inducing the most pronounced changes and differential gene expression. The clustering and functional enrichment analyses of differentially expressed genes (DEGs) highlighted the enrichment of the induced genes in pathways related to plant hormone signaling, phenylpropanoid biosynthesis, and transcription factors following stress treatments. In these pathways, the synthesis and signal transduction of abscisic acid (ABA) and ethylene, as well as the flavonoid and lignin synthesis pathways, and transcription factors such as MYB, AP2/ERF, bHLH, NAC, and WRKY responded actively to the stress and played pivotal roles. Through the WGCNA analysis, 10 key modules were identified, with the yellow module demonstrating a high correlation with the ABA and anthocyanin contents, while the turquoise module was enriched in the majority of genes related to hormone and phenylpropanoid pathways. The analysis of hub genes in these modules highlighted the significant roles of the bHLH and MYB transcription factors. These findings could offer new insights into the molecular mechanisms that enable the adaptation of *K. arborescens* to desert environments, enhancing our understanding of how other desert plants adapt to harsh conditions. These insights are crucial for exploring and utilizing high-quality forage plant germplasm resources and ecological development, with the identified candidate genes serving as valuable targets for further research on stress-resistant genes.

## 1. Introduction

Land desertification and salinization have led to significant soil erosion, constraining the development of global agriculture. The drought, salt, and alkali stresses resulting from desertification can impose severe osmotic stress, ion toxicity, and high-pH damage on plants [1,2,3,4,5], severely inhibiting their growth and development and exacerbating global ecological degradation [6,7]. Adaptation is essential for sessile plants to survive, driving the evolution of complex sensing, signaling, and stress response mechanisms under adverse conditions. These mechanisms can regulate the primary and secondary metabolic processes through hormones, transcription factors, and second messengers (such as calcium ions and reactive oxygen species), restoring cellular osmotic, ionic, and reactive oxygen species (ROS) balance, thereby enhancing plant resilience in stressful environments [3,7,8,9].

The plant hormones, such as abscisic acid (ABA), ethylene, auxin, cytokinins (CKs), gibberellin (GA), brassinosteroids (BRs), jasmonic acid (JA), and salicylic acid (SA), are crucial small-molecule regulators of plant growth. They play intricate and effective roles across different growth stages and environmental conditions, mediating plant growth, development, and adaptation to various environmental challenges [10]. The ABA signaling pathway is widely recognized as a pivotal hormone signaling pathway that is central to plant responses to abiotic stress [11]. Under such stress conditions, ABA accumulates rapidly, leading to the binding of PYLs to clade A PP2Cs. This interaction releases the inhibition of SnRK2 protein kinases and activates their phosphorylation activities. SnRK2s subsequently phosphorylate certain substrates, such as ion channel proteins, transcription factors, or transporter proteins, thereby regulating stomatal closure and maintaining homeostasis to mitigate stress-induced plant damage [12]. The ethylene synthesis and signaling pathways also play crucial roles in enhancing plant tolerance to the abiotic stress. In *Arabidopsis*, the increased ethylene levels associated with the *eto1-1* (ethylene overproducer 1) allele are associated with improved plant salt tolerance. This effect can be achieved by reducing Na^+^ influx into the roots and its transport to the shoots, thereby enhancing Na^+^/K^+^ homeostasis [13]. During drought stress, ethylene promotes stomatal closure by stimulating NADPH oxidase-mediated ROS production in the guard cells [14]. The components of the ethylene signaling pathway, such as MKK9, EIN2, and EIN3, positively regulate plant stress resistance [15,16,17,18]. Salicylic acid regulates the photosynthesis and antioxidant systems in plants under stress conditions, whereas jasmonates modulate stomatal movement and ion balance. Each of these plant hormones plays a critical role in facilitating adaptation to diverse environmental conditions [19,20].

In adverse environments, plants adjust their energy and carbon flux through primary and secondary metabolic processes [3]. Abiotic stress induces the biosynthesis of flavonoids, which act as antioxidants that mitigate the oxidative damage [21]. For instance, under salt stress, rice up-regulates UDP-glucosyltransferase genes such as *GSA1* to promote the flavonoid glycoside accumulation, reducing the oxidative damage caused by the stress [22]. Drought stress in apples induces *HSFA8a* interaction with *RAP2.12*, leading to increased expression of *MYB12*, *ANS*, and *FLS* genes and the accumulation of flavonoids, such as anthocyanins and flavanols [23]. In addition, abiotic stress prompts plants to synthesize lignin. Drought stress induces *CmCAD1*, *2*, and *3* expression in melons, enhancing lignin deposition for efficient axial water transport and improving stress resistance [24,25]. Meanwhile, genomics, transcriptomics, and metabolomic analyses of various desert plants have shown that the phenylpropane metabolic pathway can enhance the plant’s osmotic regulation ability, reactive oxygen species detoxification ability, and cell membrane stability, thereby helping the plant survive in adversity [26,27].

During the plant response to abiotic stress, transcription factor families such as MYB, AP2/ERF, NAC, and WRKY play crucial roles in the transcriptional regulation [28]. These factors primarily govern the antioxidant activity, osmotic regulation, ion transport systems, and stress response genes to maintain intracellular stability, thereby enhancing stress resistance [29,30,31]. Studies have indicated the broad regulatory impact of transcription factors on plant hormone signaling and secondary metabolite synthesis pathways. In the ethylene signaling pathway, the ERF1 transcription factor can act on EIN3. For instance, the overexpression of *SodERF3* in sugarcane enhances its resistance to salt and drought stresses [32]. In addition, the MBW ternary complex, consisting of MYB, bHLH, and WD40 proteins, plays a pivotal role in lignin and flavonoid biosynthesis [33].

Desert plants can thrive in arid, high-temperature, and saline environments and exhibit robust drought and salt resistance [34,35]. Morphologically, most desert plants have hydrotropic root systems, with the root depth and breadth several to dozens of times greater than the aboveground parts [36]. In addition, the roots, stems, and leaves demonstrate water-storage characteristics [37]. Typical features of desert plants include thickened leaf cuticles, low stomatal density, and leaves with trichomes or a reduced leaf area [38]. To minimize water evaporation, some desert plants are nearly leafless, and photosynthesis is conducted through green branches or stems, as observed in *Haloxylon species* [39]. Additionally, certain plants such as *Reaumuria trigyna* have developed unique clonal growth strategies, known as split growth, to thrive under challenging desert conditions [40]. Physiologically, halophytic plants maintain an ionic balance through salt secretion via the salt glands [41]. Recent advancements in research on stress resistance mechanisms in desert plants have progressed significantly to the molecular biology level. Genomics, transcriptomics, proteomics, and metabolomics have made substantial contributions to this field [26,27,42,43,44,45,46,47,48,49]. Studies have identified the roles of ion channel transporters, oxidation-reduction processes, transcription factors, hormone biosynthesis, and signal transduction in the adaptation of desert plants to harsh habitats [26,41,42,44,45,49,50]. Flavonoids and phenylpropanoids, particularly flavonoids and lignin, accumulate significantly under abiotic stress [15,27,43,51,52]. Various stress-resistance genes have been identified in desert plants, including *WRKYs*, *NHXs, HKTs*, and *AKTs* [53,54,55,56,57]. For instance, the co-expression of *ZxNHX* and *ZxVP1-1* genes in *Zygophyllum xanthoxylum* can enhance stress resistance and biomass in transgenic alfalfa [58]. *RtNAC055* from *Reaumuria trigyna* can improve drought tolerance in transgenic *R. trigyna* callus and poplar plants by regulating stomatal closure and maintaining antioxidant balance [59]. The *NsHKT1* gene from *Nitraria sibirica* significantly influenced the secondary metabolic pathways, the MAPK signaling pathway, and the ion regulation systems in transgenic 84K poplar [60]. Therefore, different plants exhibit unique stress adaptation features. Research on the existing stress-resistant plants in nature can significantly enrich our understanding of plant stress adaptation mechanisms and promote the development and utilization of these plant genetic resources.

*Krascheninnikovia arborescens*, a member of the Amaranthaceae family and the *Krascheninnikovia* genus, is an endemic plant of China renowned for its robust stress resistance. It features notable morphological traits such as large biomass, hairy leaves, a well-developed root system, and the ability to thrive in barren, arid, and saline-alkaline desert environments such as mountains and grasslands [61,62]. Moreover, this plant serves as a crucial forage species in desert ecosystems and has been extensively utilized for ecological restoration, indicating significant research and practical value [62,63]. Current research on *K*. *arborescens* has primarily focused on its physiological resistance and cultivation techniques, with a limited understanding of its molecular mechanisms of stress resistance [62]. Recently, a novel protein named *KaMTP* was discovered in *K*. *arborescens*, which complemented the sensitivity of yeast mutants to excessive Zn. Thus, the *KaMTP* gene is considered highly sensitive to zinc stress, indicating that the species is also a pioneer in resisting mineral stress [64]. To investigate the habitat adaptation mechanisms of *K*. *arborescens*, this study focused on *K*. *arborescens* subjected to drought, salt, and alkali stresses. Based on physiological and biochemical indices, the gene expression patterns and tissue specificity were elucidated using transcriptome analysis under various stress conditions. Furthermore, this analysis identified the related transcriptional regulatory networks, key metabolic pathways, and genes crucial for the response to abiotic stress. These findings could offer new insights into the molecular mechanisms underlying stress responses, thereby providing a foundation for the effective utilization of its genetic resources as a high-quality forage plant. Moreover, this research could provide scientific evidence supporting the restoration and enhancement of arid and saline-alkaline ecosystems.

## 2. Results

### 2.1. Morphological Changes of K. arborescens Under Drought, Salt, and Alkali Stresses

**Figure 1:**
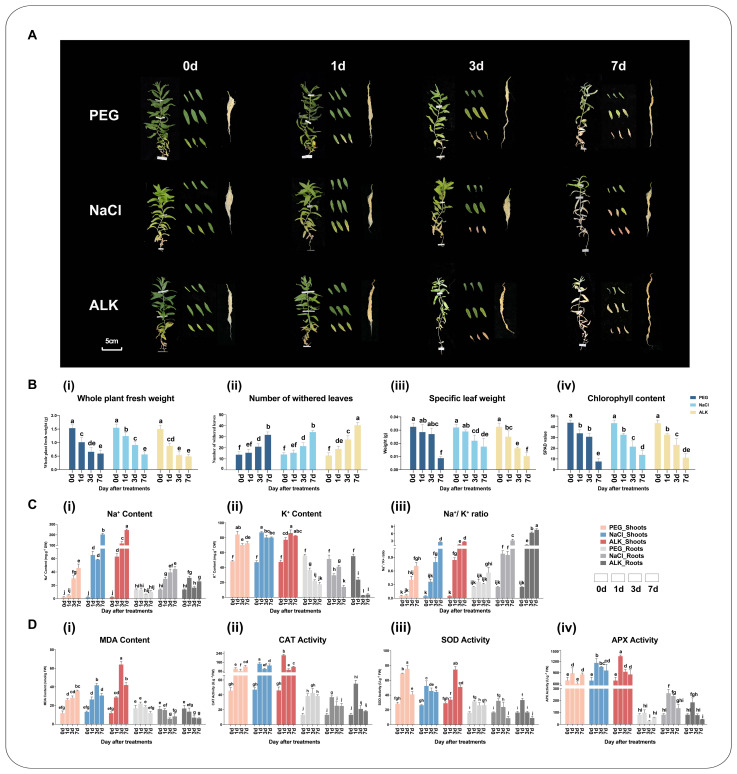
(**A**) Growth performance of *K. arborescens* under PEG 6000 treatment (PEG), NaCl treatment (NaCl), and alkaline salt treatment (ALK). Panels: Morphological changes in the aboveground parts. Morphological changes in specific leaves. Morphological changes in the roots. (**B**) Growth performance of the plants. (i) Whole plant fresh weight. (ii) Number of withered leaves. (iii) Specific leaf weight. (iv) Chlorophyll content. (**C**) Na^+^ (i) and K^+^ (ii) contents and Na^+^/K^+^ ratio (iii) in shoots and roots of plants. (**D**) MDA (i) content and activities of CAT (ii), SOD (iii), and APX (iv) in shoots and roots of plants. Bars represent the means ± SDs of three replicates. Significant differences among treatments are indicated by different letters within a panel based on Duncan’s multiple range test (*p* < 0.05).

To assess the impacts of drought, salt, and alkali stress on *K. arborescens* seedlings (hydroponically grown for approximately 55 days), stress treatments were applied with an electrolytic leakage rate approximately twice that of the control conditions (0 d, CN): 20% PEG6000 (PEG), 200 mM NaCl (NaCl), and 150 mM alkaline salt (ALK)) (Figure S1). The phenotypic and physiological biochemical indices were measured at intervals of 0 days (0 d), 1 d, 3 d, and 7 d after stress initiation (Figure 1). The results showed that CN had overall vigorous growth, lush branches and leaves, and fewer withered leaves. Most of the leaves were wide and had high chlorophyll content. The root biomass was large and appeared white or light yellow. The content of Na^+^/K^+^ ions and the activity of antioxidant enzymes were relatively low, indicating that the plant’s internal homeostasis was relatively balanced (Figure 1). Compared with the non-treated controls, the *K. arborescens* seedlings exhibited inhibited growth under drought, salt, and alkali stress conditions. This was evidenced by the gradual chlorosis and necrosis from the bottom to the top leaves, slow and browned root growth, and a significant reduction in overall biomass (Figure 1A,B). Compared to drought and salt stresses, alkali stress had the most significant impact on the seedlings, with the most pronounced PCD in the leaves (Figure 1A,B(ii)). The measurements of leaf chlorophyll content (SPAD) indicated the inhibition of photosynthesis in seedlings under different stress treatments. Additionally, the contents of MDA and Na^+^/K^+^ ions, along with the activities of antioxidant enzymes (SOD, CAT, and APX), were significantly increased (Figure 1C,D), suggesting that the *K. arborescens* seedlings experienced severe oxidative damage and osmotic stress due to the salt, alkali, and drought stress. The comprehensive analysis of phenotypic characteristics, MDA content, and Na^+^/K^+^ ratio revealed that the alkali stress caused more damage to plants than the salt and drought stress.

### 2.2. Transcriptome Sequencing and De Novo Assembly

**Figure 2:**
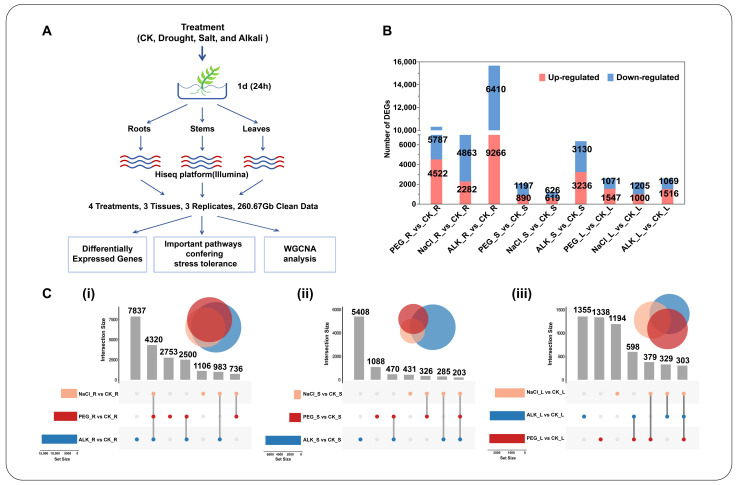
(**A**) Schematic of the experimental design. (**B**) Number of DEGs under drought, salt, and alkali treatments in roots, stems, or leaves of *K. Arborescens*. (**C**) Venn diagrams of DEGs under different treatments in roots (i), stems (ii), and leaves (iii).

When plants are subjected to environmental stress, different treatments and tissues can exhibit varied gene expression patterns, reflecting the spatiotemporal differences in their response strategies. Based on the MDA content (Figure S2), treatments with MDA content approximately double that of the non-treated controls (0 d) were selected. The samples treated under 20% PEG6000 (PEG), 200 mM NaCl (NaCl), and 150 mM alkaline salt (ALK) stress conditions for 1 d were adopted, and the root, stem, and leaf materials were collected for transcriptome sequencing. A total of 36 samples (three biological replicates for each tissue under each treatment) generated 260.67 Gb of data, averaging more than 5.92 Gb of clean reads per sample. The Q20 and Q30 scores exceeded 97.3% and 92.21%, respectively, with the GC content ranging between 41.59% and 43.96% (Figure 2A and Table S1). The de novo assembly yielded 139,176 unigenes, averaging 983.62 bp in length with an N50 of 1432 bp. These findings indicated that the quality of the transcriptome sequencing data in this study was high and suitable for subsequent analyses (Table S2).

### 2.3. Identification of Differential Expression Genes

The comparative transcriptome analysis revealed 17,884 DEGs under alkali stress, 12,446 DEGs under drought stress, and 8805 DEGs under salt stress compared to the CN group. The highest number of DEGs were identified under alkali stress, followed by drought stress, and the least under salt stress (Figure 2B). Across the roots, stems, and leaves, 20,271, 8211, and 5496 DEGs were identified, respectively, indicating significant tissue-specific gene expression patterns. A total of 10,309, 2087, and 2618 DEGs were identified in roots, stems, and leaves, respectively, in response to drought stress. In response to salt stress, 7145, 1245, and 2205 DEGs were identified in roots, stems, and leaves, respectively. For alkaline stress, 15,676, 6366, and 2585 DEGs were identified in roots, stems, and leaves (Figure 2B and Table S3). The roots exhibited a significantly higher number of DEGs than the stems and leaves, particularly under alkali stress, suggesting the early initiation of stress response regulation in the roots. The alkali stress had the most significant impact on the gene transcription in the roots and stems, while the salt stress had the least impact, which demonstrated the heightened sensitivity of *K. arborescens* to alkali stress with extensive gene expression regulation and stress response mechanisms (Figure 2C). The Venn analysis revealed more common DEGs in the roots under all three stress types (4320, 21.31%), highlighting the commonalities in the stress response mechanisms across different stress conditions in *K. arborescens* roots (Figure 2C(i)).

### 2.4. Functional Enrichment Analysis of DEGs

The cluster analysis indicated diverse expression patterns of the DEGs in the roots, stems, and leaves of *K. arborescens* under all three stress conditions. 

**Figure 3:**
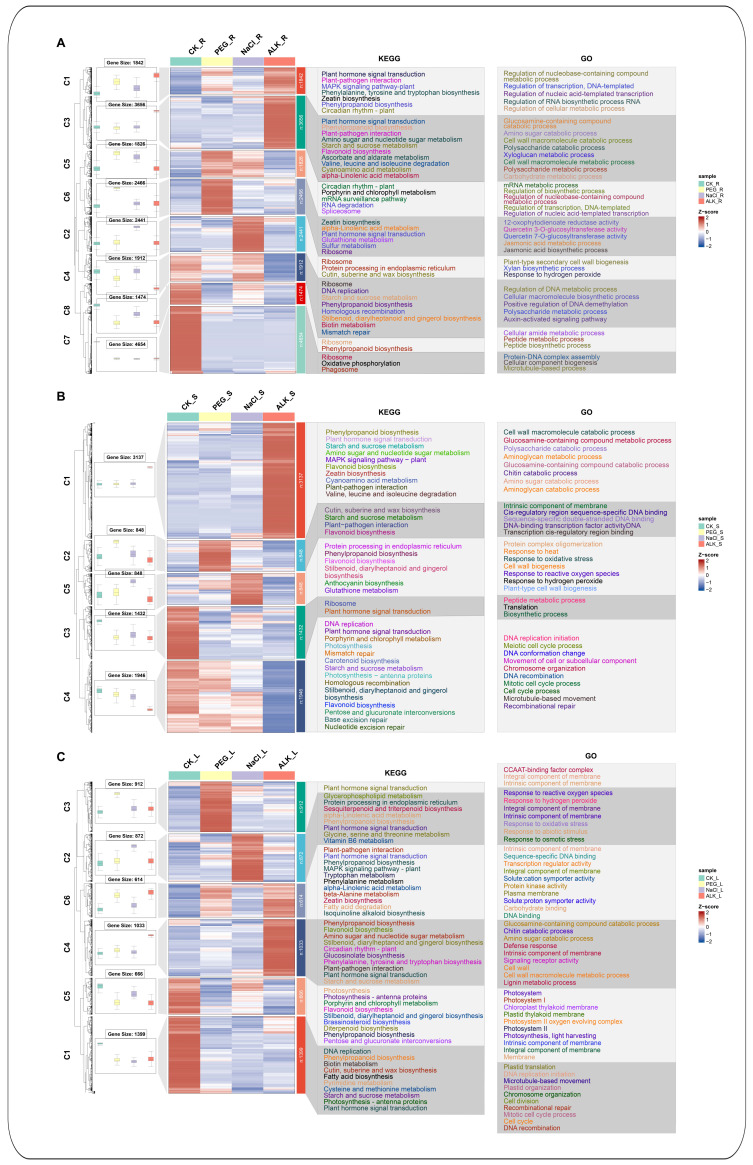
Mfuzz cluster analysis and functional enrichment analysis of DEGs under drought, salt, and alkali treatments in roots (**A**), stems (**B**), and leaves (**C**) of *K. arborescens*.

In the roots (Figure 3A), 20,271 DEGs were categorized into eight clusters. Cluster 1 comprised 1824 DEGs that were upregulated under drought and alkali stresses. Clusters 2 and 6 contained 2441 and 2466 DEGs significantly upregulated under drought and salt stresses, respectively. Cluster 3 contained 3656 DEGs that were up-regulated exclusively under the alkali stress. Cluster 4 contained 1912 genes with relatively high expression levels under the CN, drought, and salt stress conditions. Cluster 5, with 1826 genes, presented the increased expression under the drought and salt stress. Cluster 7 comprised 4554 genes with higher expression levels in the CN group. Cluster 8 included 1474 DEGs with higher expression levels under the CN and salt stress conditions. 

In the stems (Figure 3B), 8211 DEGs were grouped into five clusters. Cluster 1 comprised 3137 DEGs that were upregulated under the alkali stress. Clusters 2 and 5 included 848 DEGs upregulated under drought and salt stresses, respectively. Cluster 3 contained 1432 genes with higher expression levels in the CN group. Cluster 4, consisting of 1946 DEGs, indicated relatively high expression levels under the CN, drought, and salt treatments. 

In the leaves (Figure 3C), 5496 DEGs were classified into six clusters. Cluster 1 comprised 1399 genes with higher expression levels in the CN group. Clusters 2 (872 genes), 3 (912 genes), and 4 (1033 genes) demonstrated higher expression levels under drought, salt, and alkali stresses, respectively. Cluster 5, with 666 genes, exhibited higher expression levels under the CN and salt stress. Cluster 6, containing 614 DEGs, had higher expression levels under drought and alkali stresses.

KEGG and GO enrichment analyses revealed that the upregulated DEGs following various stress treatments were predominantly enriched in KEGG pathways, such as ‘plant hormone signal transduction’, ‘phenylpropanoid biosynthesis’, and ‘plant-pathogen interaction’, along with the GO terms including ‘DNA-binding transcription factor activity’, ‘integral component of membrane’, and ‘intrinsic component of membrane’. These findings indicated the significant roles of these metabolic pathways and cellular processes in the response of *K. arborescens* to different abiotic stressors (Figure 3 and Tables S4 and S5).

### 2.5. DEGs Related to Hormone Biosynthetic and Signal Transduction Pathways and Their Expression Profiling Under Abiotic Stress

In the eight hormone pathways, a total of 256 DEGs encoding 59 enzymes were identified, comprising 140 DEGs associated with the stress response hormones (ABA, ethylene, SA, and JA) and 118 DEGs related to the growth promotion hormones (auxin, GA, CKs, and BRs) (Table S6 and Figure S3).

**Figure 4:**
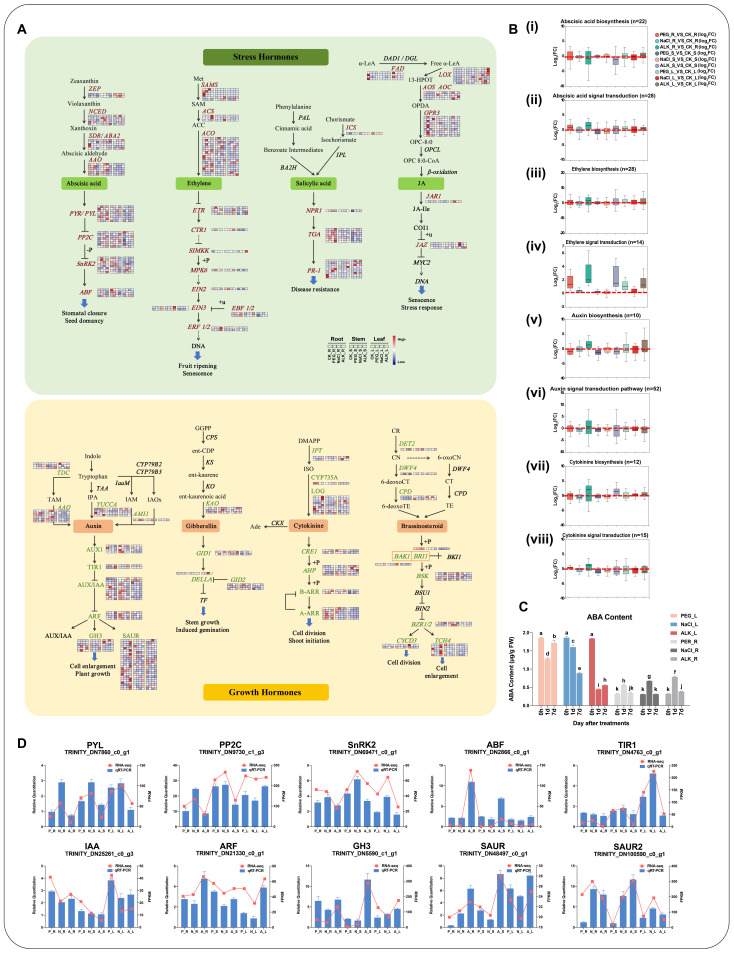
Hormone synthesis and signal transduction pathways. (**A**) Schematic diagrams of hormone synthesis and signal transduction pathways. DEGs are marked in color. The heatmap color indicates the FPKM value. (**B**) Expression profiles of genes involved in abscisic acid and ethylene synthesis and signal transduction pathways ((i), (ii), (iii), and (iv)), and the auxin and cytokinine synthesis signal transduction pathway ((v), (vi), (vii), and (viii)). (**C**) ABA content under different treatments. Bars represent the means ± SDs of three replicates. Significant differences among treatments are indicated by different letters within a panel based on Duncan’s multiple range test (*p* < 0.05). (**D**) Transcriptional analysis and qRT-PCR assays of 10 genes related to abscisic acid and auxin signal transduction.

In the stress hormone-related pathways, the gene expression levels generally exhibited the upward trends across all treatment groups. Compared with CN, the average FC value of each treatment group reached 22.72, with the most significant increase in the gene expression observed under the alkaline stress. Specifically, compared with CN, the average FC values for genes in the ALK_R, ALK_S, and ALK_L groups were 41.15, 23.62, and 56.95, respectively (Table S6). These pathways featured a higher number of DEGs associated with the ABA and ethylene biosynthesis and signal transduction. In different tissues under drought, salt, and alkali stress, 50 DEGs related to ABA were identified, eight of which were significantly up-regulated in every treatments, primarily including the NCED (TRINITY_DN38279_c0_g1), PYL (TRINITY_DN7860_c0_g1), PP2C (TRINITY_DN9730_c1_g3, TRINITY_DN9148_c0_g1, TRINITY_DN10220_c0_g1, TRINITY_DN4606_c0_g1, and TRINITY_DN30473_c0_g1), and ABF (TRINITY_DN2866_c0_g1) genes. Notably, three PP2C-encoding genes exhibited higher expression levels, averaging 97.78 FPKM across the treatment groups and reaching 190.31 FPKM under the alkali stress. Specifically, TRINITY_DN9730_c1_g3 exhibited the significant up-regulation, with the log2FC ranging from 2.60 to 7.28 and averaging 4.48. The ABF-encoding gene, TRINITY_DN2866_c0_g1, also displayed the high expression levels (FPKM more than 100), averaging 92.96 FPKM across the treatment groups (Table S6). In the ethylene biosynthesis and signal transduction pathways, 42 DEGs were identified, 10 of which were significantly induced across different tissues under all the stresses. These included ethylene biosynthesis-related genes, such as SAMS (TRINITY_DN35855_c1_g2) and ACO (TRINITY_DN333_c1_g2 and TRINITY_DN8359_c0_g1), and ethylene signal transduction-related genes, such as ETR (TRINITY_DN21313_c0_g2), SIMKK (TRINITY_DN4081_c0_g1), MPK6 (TRINITY_DN10111_c0_g1), EIN3 (TRINITY_DN2770_c0_g1 and TRINITY_DN2176_c0_g1), and two EBF1/2 (TRINITY_DN160352_c0_g1 and TRINITY_DN10712_c0_g1). Among them, the genes of SAMS, ACO, and EIN3 demonstrated the relatively high expression levels, averaging 200.41 FPKM for 5 genes across the treatment groups. Specifically, the SAMS gene TRINITY_DN35855_c1_g2 exhibited the significant up-regulation, with an average FPKM of 629.71, particularly under the alkali treatment. Conversely, fewer DEGs were related to the SA and JA pathways, including only SA signal transduction-related genes TGA and PR-1 and JA biosynthesis-related genes LOX and OPR3 (Figure 4A and Figure S3).

In the growth hormone pathways, most DEGs exhibited the down-regulated expression across various tissues under the drought, salt or alkali stress conditions. However, under the alkali stress, most genes were up-regulated, notably with the significant increase in expression observed in the stems. Under alkali stress, compared to the CN group, the average FC value was 18.49 in the stems, 10.06 in the roots, and 9.14 in the leaves (Figure 4A and Figure S3 and Table S6). Among the Growth hormones pathways, the auxin pathway had the highest number of DEGs identified, followed by the CKs, BRs, and GA pathways. In the auxin biosynthesis and signal transduction pathways, 62 DEGs were identified, 9 of which were downregulated under different stresses. These included AUX1 (TRINITY_DN84076_c0_g1 and TRINITY_DN36620_c0_g1), IAA (TRINITY_DN1714_c0_g2, TRINITY_DN1649_c0_g2, and TRINITY_DN37896_c0_g2), ARF (TRINITY_DN188745_c0_g1), GH3 (TRINITY_DN9389_c0_g1), and SAUR (TRINITY_DN14664_c0_g1 and TRINITY_DN7691_c0_g1) genes. Notably, TRINITY_DN37896_c0_g2 and TRINITY_DN84076_c0_g1 exhibited higher expression levels and were significantly downregulated across different treatment groups, particularly in the roots and stems under the alkali stress, with an average log_2_FC value of −5.66. In the CKs pathway, 27 DEGs were identified, with only one gene encoding AHP (TRINITY_DN58819_c1_g2) consistently downregulated under all treatments. However, its expression level was low, averaging only 1.79 FPKM in the treatment groups.

We validated the the randomly selected hormone related genes expression results from RNA-seq by RT-qPCR and found both results consistent (Figure 4D and Table S11). Subsequently, the measurements of ABA content in the leaves and roots of *K. arborescens* under the drought, salt, and alkali stress treatments at 0 d, 1 d, and 7 d demonstrated the decreases in the leaf ABA content, most notably under the alkaline stress. Initially, the root ABA content was significantly lower than that in the leaves at 0 d, but increased markedly after 1d under various stress treatments, particularly under the alkali stress, returning to initial levels after 7 d. The transcriptome analysis revealed the increased expression of DEGs related to the ABA synthesis and signaling pathways in the roots (mean FC value = 4.27) than in leaves (mean FC = 2.66), corroborating these findings (Figure 4C). This suggested the more pronounced response of *K. arborescens* to the alkali stress, which was characterized by the heightened involvement of the stress hormone-related genes, particularly those involved in the ABA synthesis and signaling pathways, compared to the salt and drought stress.

### 2.6. DEGs Related to Phenylpropanoid Metabolic Pathways and Their Expression Profiling Under Abiotic Stress

In the phenylpropanoid metabolic pathway, 97 DEGs encoding 18 related enzymes were identified (Table S7). 

**Figure 5:**
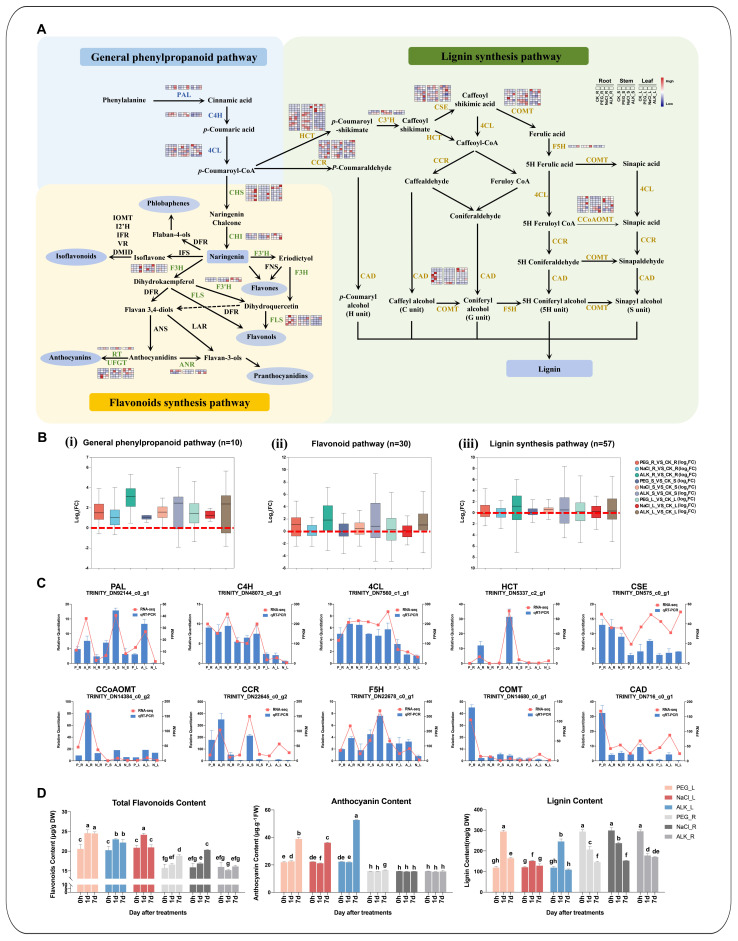
Phenylpropanoid and flavonoid biosynthesis pathways. (**A**) Schematic diagrams of phenylpropanoid and flavonoid biosynthesis. DEGs are marked in color. The heatmap color indicates the FPKM value. (**B**) Expression profile of genes involved in the general phenylpropanoid pathway (i), flavonoid synthesis pathway (ii), and lignin synthesis pathway (iii). (**C**) Transcriptional analysis and qRT-PCR assays of 10 genes related to phenylpropanoid and flavonoid biosynthesis. (**D**) Contents of flavonoids, anthocyanin, and lignin under different treatments. Bars represent the means ± SDs of three replicates. Significant differences among treatments are indicated by different letters within a panel based on Duncan’s multiple range test (*p* < 0.05).

Specifically, 10 DEGs were found in the general phenylpropanoid pathway, including the PAL, C4H, and 4CL encoding genes. Among them, four DEGs were up regulated in the roots, stems, and leaves under the drought, salt or alkali stress, exhibiting high expression levels (FPKM more than 100). These genes were TRINITY_DN4885_c1_g1 encoding PAL, TRINITY_DN6742_c1_g2 and TRINITY_DN48073_c0_g1 encoding C4H, and TRINITY_DN7560_c1_g1 encoding 4CL. Notably, TRINITY_DN4885_c1_g1 demonstrated the exceptionally high expression in the roots, stems, and leaves under the alkali stress, with the FPKM of 1023.73 in ALK_R, 926.88 in ALK_S, and 690.44 in ALK_L (Figure 5B(i)). These findings indicated that the phenylpropanoid metabolic pathway was actively responsive to the stresses, facilitating the production of precursor substances essential for the downstream flavonoid and lignin formation.

In the flavonoid synthesis pathway, 30 DEGs were identified, including the up-regulated CHS (TRINITY_DN3711_c0_g1), CHI (TRINITY_DN873_c1_g1), F3H (TRINITY_DN3561_c1_g1), and UFGT (TRINITY_DN1370_c1_g1) genes in all treatment groups. Notably, TRINITY_DN3561_c1_g1 and TRINITY_DN3711_c0_g1 exhibited the most significant up-regulation under the alkali stress, with the average FPKM values being 6.01 times higher than other stress treatments.

Among the 57 DEGs related to the lignin synthesis pathway, 12 were highly expressed. These included the CCR (TRINITY_DN22645_c0_g2 and TRINITY_DN415_c0_g1), C3H (TRINITY_DN5259_c0_g1), CSE (TRINITY_DN6419_c0_g1), COMT (TRINITY_DN14680_c0_g1, TRINITY_DN8536_c1_g1, and TRINITY_DN10175_c0_g1), F5H (TRINITY_DN22678_c0_g1), CCoAOMT (TRINITY_DN14384_c0_g2 and TRINITY_DN1379_c0_g1), and the CAD genes (TRINITY_DN55848_c0_g1 and TRINITY_DN716_c0_g1). Notably, TRINITY_DN22645_c0_g2 (CCR), TRINITY_DN5259_c0_g1 (C3H), TRINITY_DN22678_c0_g1 (F5H), and TRINITY_DN716_c0_g1 (CAD) exhibited the particularly high expression levels and were upregulated in the roots, stems, and leaves under various stress treatments, particularly under the alkali stress, where their average FPKM were 1.76 times higher than other stress treatments.

Randomly select 10 genes related to phenylpropane metabolism for RT-qPCR to verify RNA-seq results and find that the two results are consistent (Figure 5D and Table S11). The measurements of the total flavonoid, anthocyanin, and lignin contents in the leaves and roots of *K. arborescens* under the drought, salt, and alkali stress revealed the increased accumulation of these substances in the leaves under all three stress conditions. The highest levels were observed under the alkali stress, consistent with the higher expression levels of previously identified DEGs related to the flavonoid and lignin synthesis under the alkali stress (the average FC value being 8.88 times higher than that under other stresses). This finding indicated that the phenylpropanoid metabolites, such as the total flavonoids and lignin, played crucial roles in the response of *K. arborescens* to the abiotic stress.

### 2.7. Transcription Factors Identifying and Their Expression Profiling Under Abiotic Stress

From the transcriptome databases under the three stress conditions, 1200 coding genes from 33 transcription factor families were identified. Among these, 408 genes exhibited differential expression across different stress conditions and were categorized into 31 transcription factor families (Table S8).

The ten transcription factor families with the most DEGs were MYB, AP2/ERF, C2C2, bHLH, NAC, bZIP, B3_superfamily, WRKY, C3H, and GRAS (Figure S4). Among them, the MYB, AP2/ERF, and C2C2 families, which had the highest number of DEGs, contained 58, 51, and 36, respectively. These genes exhibited the average FC values of 2.46 under the drought stress, 6.33 under the salt stress, and 8.23 under the alkali stress, indicating their active responses to these stresses. Notably, six MYB genes (TRINITY_DN2434_c0_g1, TRINITY_DN2151_c0_g1, TRINITY_DN561_c0_g1, TRINITY_DN392_c1_g2, TRINITY_DN18689_c0_g1, and TRINITY_DN63812_c0_g1), two C2C2 genes (TRINITY_DN3191_c0_g1 and TRINITY_DN32779_c1_g1), and one AP2/ERF gene (TRINITY_DN7940_c3_g1) were up-regulated across different treatment groups.

Among the transcription factor families, the NAC and WRKY families collectively comprised 55 DEGs, exhibiting the average FC value of 7.52 under various stress treatments, indicating a general trend of up-regulation (Figure S4). Specifically, the WRKY-coding genes (TRINITY_DN2552_c0_g1, TRINITY_DN1057_c0_g1, and TRINITY_DN8219_c0_g1), along with the NAC-coding genes (TRINITY_DN1120_c1_g1, TRINITY_DN2033_c0_g1, TRINITY_DN41540_c0_g1, TRINITY_DN1073_c0_g1, TRINITY_DN12955_c0_g1, TRINITY_DN4556_c0_g2, TRINITY_DN3721_c0_g1, and TRINITY_DN20288_c0_g2) were up-regulated across different treatment groups. Notably, TRINITY_DN2552_c0_g1, TRINITY_DN1120_c1_g1, TRINITY_DN2033_c0_g1, TRINITY_DN41540_c0_g1, and TRINITY_DN1073_c0_g1 exhibited particularly high expression levels, especially under the alkali stress, where the average FPKM reached 140.42 (Figure S4).

### 2.8. Mining of Abiotic Stress-Responsive Genes by WGCNA Analysis

**Figure 6:**
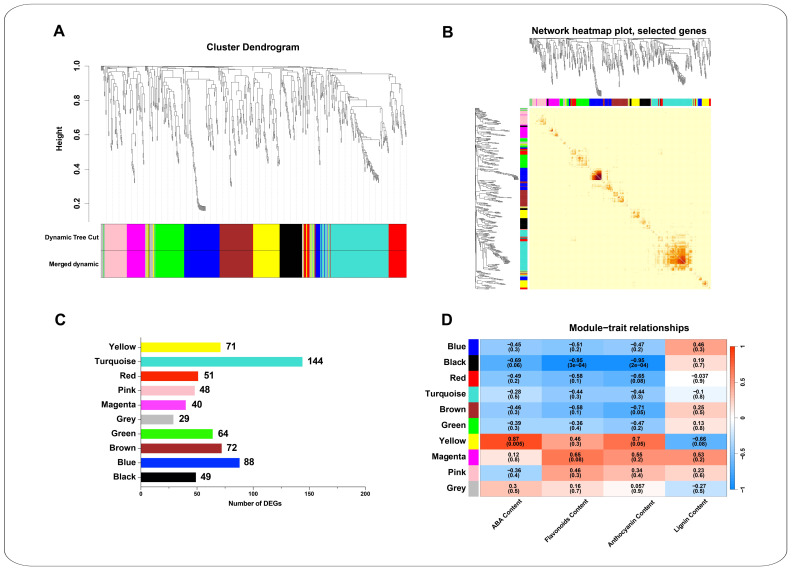
Weighted gene co-expression network analysis (WGCNA) of DEGs. Cluster Dendrogram (**A**) Hierarchical clustering tree showing the co-expression modules identified by WGCNA. Different modules are marked in different colors. Each leaf in the cluster tree represents a gene. Network heatmap plot of selected genes (**B**) Network heatmap of module genes. Each tree represents a module, and each branch represents a gene. The darker the color of each dot, the stronger is the connectivity between the two genes in the row and column. (**C**) Number of DEGs assigned to different modules. Module-trait relationships (**D**) Correlations between modules and dominating plant hormone component traits in *K. arborescens*.

The Weighted Gene Co-expression Network Analysis (WGCNA) was conducted to elucidate the transcriptional regulatory relationship of the hormone biosynthetic and signal transduction pathways and phenylpropanoid metabolic pathways of *K. arborescens* under the abiotic stress. This analysis utilized the DEGs from these pathways and focused on the top 10 transcription factor families associated with these DEGs. The analysis of the 656 genes presented 10 co-expression modules, such as the black module (49 DEGs), the blue module (88 genes), the brown module (72 genes), the green module (64 genes), the grey module (29 genes), the magenta module (40 genes), the pink module (48 genes), the red module (51 genes), the turquoise module (144 genes), and the yellow module (71 genes) (Figure 6A–C and Table S9). Notably, the yellow module exhibited strong correlations with the changes in ABA contents (*p* = 0.005, cor = 0.87) and anthocyanin content (*p* = 0.05, cor = 0.7) under the CN group, drought, salt, and alkali stresses (Figure 6D). Furthermore, the turquoise module was notably enriched, with a greater number of genes related to the hormone biosynthetic and signal transduction pathways and phenylpropanoid metabolic pathways.

**Figure 7:**
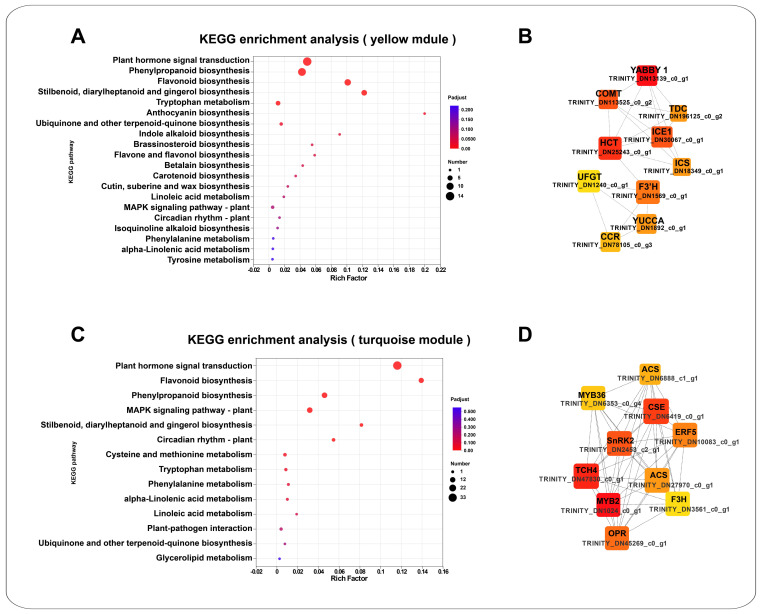
(**A**) KEGG enrichment analyses of the yellow co-expression module genes recognized by the WGCNA analysis. (**B**) Co-expression network diagram of the interaction between hub genes in the yellow module. (**C**) KEGG enrichment analyses of the turquoise co-expression module genes recognized by the WGCNA analysis. (**D**) Co-expression network diagram of the interaction between hub genes in the turquoise module.

The yellow module, which has a strong correlation with ABA content (*p* = 0.005, cor = 0.87) and anthocyanin content (*p* = 0.05, cor = 0.7), contained 71 genes (Figure S5A). These genes were primarily associated with the plant hormone signal transduction, phenylpropanoid biosynthesis, flavonoid biosynthesis, stilbenoid, diarylheptanoid, gingerol biosynthesis, tryptophan metabolism, and anthocyanin biosynthesis (Figure 7A). Among the 35 transcription factors included in this module, the key families included the bHLH (6), B3_superfamily (5), NAC (4), C2C2 (4), AP2/ERF (4), WRKY (3), bZIP (2), C3H (1), and MYB (1). The co-expression network revealed 688 pairs of edges. The MCC analysis highlighted ten genes within the yellow module with broad involvement in the plant response to the abiotic stress, including the TRINITY_DN13139_c0_g1 (C2C2), TRINITY_DN25243_c0_g1 (HCT), TRINITY_DN113525_c0_g2 (COMT), TRINITY_DN30067_c0_g1 (bHLH), TRINITY_DN1569_c0_g1 (F3′H), TRINITY_DN18349_c0_g1 (ICS), TRINITY_DN196125_c0_g2 (TDC), TRINITY_DN1892_c0_g1 (YUCCA), TRINITY_DN78105_c0_g3 (CCR), and TRINITY_DN1240_c0_g1 (UFGT) (Figure 7B). Given their strong correlation with the ABA and anthocyanin contents, these genes could play crucial regulatory roles in the ABA synthesis and signaling, as well as the anthocyanin synthesis in *K. arborescens* (Figure 6D).

The turquoise module contained 144 genes, making it the module with the most enriched genes related to hormone biosynthetic and signal transduction pathways and phenylpropanoid metabolic pathways (Figure S5B). These genes were primarily involved in the plant hormone signal transduction, the flavonoid biosynthesis, the phenylpropanoid biosynthesis, the MAPK signaling pathway, and the plant, stilbenoid, diarylheptanoid, and gingerol biosynthesis pathways (Figure 7C). The 78 transcription factors included in this module were mainly the MYB (20), AP2/ERF (12), NAC (10), WRKY (8), bZIP (8), B3 (7), bHLH (4), GRAS (4), C2C2 (4), and C3H (1). Among these DEGs, 5485 pairs of co-expression edges were linked. After performing the MCC analysis, the top ten genes were identified, such as two MYB genes (TRINITY_DN1024_c0_g1 and TRINITY_DN6353_c0_g4), one TCH4 gene (TRINITY_DN47830_c0_g1), one CSE gene (TRINITY_DN6419_c0_g1), one SnRK2 gene (TRINITY_DN2453_c2_g1), one OPR gene (TRINITY_DN45269_c0_g1), one AP2/ERF gene (TRINITY_DN10083_c0_g1), two ACS genes (TRINITY_DN27970_c0_g1 and TRINITY_DN6888_c1_g1), and one F3H gene (TRINITY_DN3561_c0_g1) (Figure 7D) (Table S10). Notably, TRINITY_DN6419_c0_g1 exhibited the high expression levels. Given the enrichment of the hormone and phenylpropanoid pathways among these genes, they were suggested to play pivotal roles in the response of *K. arborescens* to the abiotic stress.

## 3. Discussion

In arid and saline-alkaline desert environments, perennial wild grasses have developed intricate physiological and molecular mechanisms to survive against adverse conditions. Among these species, *K. arborescens*, a native grass in China, can serve as a crucial forage plant for livestock such as sheep, horses, and camels in desert grasslands and has been widely employed in ecological restoration efforts, holding substantial research and practical value [62]. Exploring the transcriptional regulatory mechanisms of different tissues in response to various environmental stresses through transcriptome analyses can be crucial for the comprehension of the habitat adaptation mechanisms of *K. arborescens* and for identifying stress-resistant gene resources. Therefore, based on the determination of physiological and biochemical indices, this study analyzed the transcriptomic characteristics of *K. arborescens* under drought, salt, and alkali stress through the transcriptome analysis.

Drought and salinization in desert environments primarily induces osmotic stress, ion toxicity, and elevated pH, thereby affecting the plant physiology [1,3,4,5]. Research on *Puccinellia tenuiflora* and other wild plants has suggested that the alkali stress exerts a more profound impact on plant growth and development than singular stressors, such as drought or salt stress, which can be consistent with the findings of this study [1,65]. This study observed the most significant values in PCD in *K. arborescens* seedlings under the alkali stress, indicating heightened oxidative damage and osmotic stress compared with that under drought and salt stress conditions (Figure 1). The transcriptome analysis revealed a higher number of DEGs in various tissues of *K. arborescens* under alkali stress than under drought and salt stress (Figure 2B). Furthermore, the roots exhibited the most sensitive response among the plant parts to all three stresses, especially the alkali stress, surpassing the stems and leaves in their reaction (Figure 2B). Under abiotic stress conditions, increasing water absorption through roots is an important strategy for plants to adapt to abiotic stress [6,66]. *K. arborescens*, thriving in desert habitats, featured a robust root system, with the subterranean part extending approximately 1.4 times the length of the above-ground portion [62]. This indicated the sensitivity of the root system to the abiotic stress in *K. arborescens*, which was crucial for maintaining the internal equilibrium under adverse conditions to sustain plant growth.

### 3.1. Stress Hormone Biosynthetic and Signal Transduction-Associated DEGs Contributed to the Response of K. arborescens in Harsh Environments

During plant resistance to abiotic stresses such as drought and salinity, hormones could act as crucial signaling molecules [67]. ABA, ethylene, SA, and JA were recognized as stress response hormones, whereas auxin, GA, CKs, and BRs were growth promotion hormones [68,69]. The transcriptomic analysis of poplar under low temperatures and salt stress revealed the enrichment of DEGs, primarily in the hormone signal transduction or biosynthesis pathways [42,50]. Similarly, in *Ammopiptanthus mongolicus*, plant hormones played a pivotal role in its adaptation to the desert environments. Our study demonstrated the prominence of stress hormone biosynthesis and signal transduction pathways in *K. arborescens* in response to abiotic stresses, with stress hormone-related DEGs significantly up-regulated, particularly in the ABA and ethylene pathways.

Under abiotic stresses, the stress signals stimulated the plants to produce ABA, which led to a rapid increase in the endogenous ABA levels [3]. This hormone regulated the intracellular osmotic pressure, stomatal movement, and plant development through the “core” ABA signaling pathway, thereby enhancing the water-use efficiency and mitigating the osmotic stress damage [5,6,12,70]. Under salt stress, the KEGG pathway with the highest enrichment of DEGs in *Solenostemma argel* roots and leaves is the plant hormone signal transduction pathway, and the ABA pathway was significantly enriched in both samples [71]. In *Tartary buckwheat*, three ABA biosynthesis genes (one ZEP/ABA1 and two NCED3) and eight ABA signaling genes (one PYL/PYR, four PP2C, one SnRK2.6/OST1, and two AREB/ABF) play a central role in its drought resistance process [72].

Similarly, our study revealed the significant up-regulation of the NCED, PYL, PP2C, and ABF genes in the ABA biosynthesis and signal transduction pathways under drought, salt, and alkali stress conditions. The NCED catalyzed the initial step of the ABA biosynthesis [73]. In the transcriptome of the salt-stressed poplar, two NCED genes (POPTR_0011s11370.1 and POPTR_0001s40420.1) were up-regulated [42]. The overexpression of NCED genes in plants led to increased accumulation of ABA and enhanced the resistance to drought, salt, and water stress [74,75,76]. PYL served as the primary receptor for ABA and, in conjunction with ABA, regulated the activity of PP2Cas [77]. In *Arabidopsis* stomata, *AtPYL4* responded to the ABA signals, maintaining water loss rates by controlling the stomatal conductance, thereby enhancing drought resistance in plants [78]. Studies on *Gossypium hirsutum* have highlighted the pivotal role of *GhPYL8D2* in responding to drought stress through co-regulating the stomatal conductance with *GhHAI2D* [79]. The A-type PP2Cs (ABI1, ABI2, HAB1, and PP2CA), as core participants downstream in the signal transduction pathway, could control stomatal opening and closing and antioxidant enzyme activity, thereby regulating the stress response process of the plants [80,81,82,83]. Additionally, PP2Cs constituted the largest protein phosphatase family in plants, comprising 76 members and demonstrating considerable complexity [84]. Studies revealed that the expression patterns of PP2C genes under cold stress in poplar and drought stress in peanut and strawberry were consistent with those observed in this study (TRINITY_DN10220_c0_g1, TRINITY_DN4606_c0_g1, and TRINITY_DN9730_c1_g3), exhibiting up-regulation under stress conditions [50,85,86]. Specifically, the PP2C gene TRINITY_DN9730_c1_g3 in *K. arborescens* exhibited a notable up-regulation trend, with an FC value of 155.65 in the ALK_R group. Under osmotic stress, ABF served as the downstream effector protein that co-regulated the expression of the ABRE-dependent genes (LEA, PP2C, and various stress-responsive transcription factors) in the ABA signal transduction pathway [87,88]. The significant increase in the ABA content in the roots of *K. arborescens* under the treatments indicated the activation of the ABA biosynthesis and signal transduction pathways, which were crucial for regulating the intracellular osmotic stresses and stomatal movements to enhance water-use efficiency and adaptability to adverse stress environments. 

Similar to ABA, the ethylene content accumulated under abiotic stress and subsequently regulated plant responses to these conditions [89,90,91,92]. In desert plants, such as *Rhazya stricta* and *Solenostemma argel*, the synthesis and signaling pathways of ethylene were crucial for their adaptation to the harsh habitats, highlighting the active roles of genes such as *ETR*, *EIN3*, and *ERF1* [71,93]. In this study, the key components of the ethylene pathway responded actively to three types of abiotic stress, with *SAMS*, *ACO*, and *EIN3* being up-regulated, reaching an average FPKM of 200.41 in the treatment groups. Among them, the gene TRINITY_DN35855_c1_g2 encoded the ethylene synthase SAMS, which exhibited the most significant up-regulation trend under the stress conditions, particularly under alkali stress, with an average log_2_FC of 8.36. During the ethylene synthesis, SAMS and ACO respectively catalyzed the formation of S-adenosyl-methionine (S-AdoMet) and the subsequent formation of the ethylene precursor 1-aminocyclopropane-1-carboxylate (ACC) [94]. SAMS was responsible not only for the SAM synthesis but also for regulating the plant-environment interactions [95,96]. The overexpression of the *SAMS1* gene enhanced the tolerance of tobacco to drought and salt stress; the stress treatments amplified the effects of the *SAMS1* gene on polyamine and ACC synthesis, rendering the transgenic lines more resilient to the adverse conditions [95]. ACO (ACC oxidase), one of the rate-limiting enzymes in ethylene synthesis, was activated under salt stress to participate in the regulation of the salt stress response [90,97]. Downstream in the ethylene signaling pathway, the EIN3 and AP2/ERF transcription factors were crucial in the stress response of plants [98,99]. In *Arabidopsis* lines with overexpressed *EIN3*, the transcription levels of drought- and salt-responsive genes and stress tolerance were both enhanced [100,101]. Similarly, under stress conditions, the ERF genes regulated the drought and salt tolerance by influencing the stress-responsive gene transcription and amplifying the MAPK cascade signals [102,103]. For instance, the overexpression of *PdbERF1* in wild-type *Populus* significantly enhanced drought tolerance, demonstrating higher photosynthetic rates, reduced transpiration rates, stomatal conductance, and improved water-use efficiency [104]. This suggested that certain genes such as SAMS, ACO, and EIN3 could regulate ethylene synthesis in *K. arborescens*, thereby contributing to its abiotic stress response.

### 3.2. Phenylpropanoid Metabolism-Related DEGs Contributed to the Response of K. arborescens in Harsh Environments

The phenylpropanoid metabolic pathway was vital for the secondary metabolic pathways in plants [21]. By up-regulating the expression of the genes associated with the phenylpropanoid synthesis, plants accumulated the flavonoids, lignin, and phenolic compounds, which were pivotal for plant growth, development, and defense against adverse environments [21,105,106]. In certain plants, such as *Ammopiptanthus mongolicu*, *Glycyrrhiza uralensis*, *Fagopyrum cymosum*, and *Thymus mongolicus*, the phenylpropanoid metabolites served as medicinal, spice, or essential oil components, withstanding biotic and abiotic stresses [43,51,52]. The untargeted metabolomics analyses of desert plants *Haloxylon ammodendron* and *Haloxylon persicum* under drought stress revealed enhanced osmotic regulation, reactive oxygen species detoxification, and cell membrane stability through pathways such as ‘Flavonoid biosynthesis (map00941)’ and ‘Phenylpropanoid biosynthesis (map00940)’ [27]. Studies on *Puccinellia tenuiflora* and *Camellia vietnamensis* have demonstrated the role of lignin and flavonoid biosynthesis in enhancing drought- or salt-stress tolerance [26,107]. The transcriptome and secondary metabolite analyses in this study highlighted the critical involvement of the phenylpropanoid metabolism in *K. arborescens* in response to abiotic stresses.

In the phenylpropanoid pathway, PAL, C4H, and 4CL catalyzed the essential steps, providing precursors for both flavonoid and lignin synthesis pathways [108]. These enzymes have been reported to be crucial for plant growth, development, and stress resilience due to influencing the formation of lignin and other phenylpropanoid derivatives, which may also lead to certain symptoms, such as anther sterility and seedling dwarfism [109,110,111,112]. In this study, significant up-regulation of PAL, C4H, and 4CL genes under stress conditions was observed, with an average FC of 6.54 compared to the CN. PAL (TRINITY_DN4885_c1_g1) was up-regulated by approximately 2.59-fold under drought and salt stress and 6.53–16.83-fold under alkali stress. This indicated the activation of the phenylpropanoid pathway in *K. arborescens* under the abiotic stresses, facilitating the increased synthesis of the flavonoids and lignin precursors through the enhanced activity of PAL, C4H, and 4CL enzymes. Similar findings in plants such as *Astragalus membranaceus* and *Gossypium hirsutum* suggested that their tolerance to saline and drought conditions correlated with the up-regulation of these genes [113,114].

CHS served as the initial enzyme in the flavonoid biosynthesis, directing the metabolic flow into the flavonoid pathway [21]. The downstream CHI catalyzed the conversion of chalcones to flavanones such as naringenin [21]. Studies have indicated that low temperatures induced *CHS*, *CHI*, and *FLS* gene expressions, promoting flavonoid accumulation and aiding the *Arabidopsis thaliana* in adapting to cold environments [115]. In alfalfa and *Arabidopsis thaliana*, the up-regulation of *F3H* enhanced the drought tolerance by enhancing anthocyanin biosynthesis [116,117,118]. UFGT was the key enzyme in the anthocyanin biosynthesis pathway of flavonoids, which catalyzed the glycosylation of unstable anthocyanidins to produce stable anthocyanins [21]. The inhibition of DFR and UFGT expression reduced anthocyanin biosynthesis in grapes [119]. Under diverse stress conditions, the expression levels of the *K. arborescens* genes CHS (TRINITY_DN3711_c0_g1), CHI (TRINITY_DN873_c1_g1), F3H (TRINITY_DN3561_c1_g1), and UFGT (TRINITY_DN1370_c1_g1) significantly increased, actively aiding the response of plants to adverse environments. Concurrently, the total flavonoid and anthocyanin content in leaves markedly increased, demonstrating the enhanced adaptation of *K. arborescens* to harsh desert conditions by regulating flavonoid synthesis and reducing ROS accumulation.

Lignin was related to the composition of the plant secondary cell walls, providing mechanical support, impermeability, and resistance to biodegradation [120,121,122]. Studies on certain plants such as *Sesuvium portulacastrum*, maize, and tea trees have indicated that lignin can enhance the tolerance to salt and drought stress [62,123,124]. In this study, the lignin content in *K. arborescens* leaves significantly increased under stress conditions. The expression levels of lignin synthesis-related genes (*CCRs*, *C3Hs*, *F5Hs*, and *CADs*) were notably elevated across various stress treatments, particularly under alkaline stress. This indicated the crucial role of lignin in the responses of *K. arborescens* to abiotic stresses. The up-regulation of lignin synthesis genes such as C4H, C3H, CAD, F5H, HCT, 4CL, COMT, and CCR promoted lignin deposition and the thickening of the secondary cell walls, improving plant tolerance to salt and osmotic stresses in species such as apple [125,126]. Similarly, increasing the *PAL* and *CAD* expression in *Miscanthus* and the *4CL1* expression in loquat fruit enhanced the lignin accumulation, improving plant adaptation to cold environments [127,128]. The lignin synthesis genes identified in this study exhibited higher expression levels under stress conditions, with the FPKM values exceeding 500 for the genes encoding CCoAOMT (TRINITY_DN12535_c0_g1 and TRINITY_DN1379_c0_g1) and COMT (TRINITY_DN8536_c1_g1 and TRINITY_DN10175_c0_g1) in the leaves and stems of the stressed plants. Under the alkali stress, the FPKM value of TRINITY_DN12535_c0_g1 increased significantly to 966.36, suggesting that inducing lignin synthesis-related genes could be a key strategy for *K. arborescens* to withstand adverse conditions.

### 3.3. Transcription Factor-Related DEGs Participated in the Response of K. arborescens in Harsh Environments

The transcription factors were pivotal in plants, regulating the stress adaptation and responses to environmental challenges critical for survival and continuity [30]. Families such as MYB, AP2/ERF, NAC, and WRKY played essential roles in regulating the plant-specific response processes [28]. The transcriptome analyses of resilient plants such as *Rhazya stricta*, *Cerasus humilis*, and *Pinus elliottii* highlighted the involvement of these families in responding to adverse environments, potentially by modulating hormone and terpene metabolism [93,129,130]. Similarly, in a study of *Agriophyllum squarrosum* (L.) Moq., a comprehensive analysis of the transcriptome and flavonoid-targeted metabolome was conducted, combined with WGCNA, and it was found that among the modules closely related to flavonoids, more transcription factors such as MYB, bHLH, and WD domain repeat (WDR) were involved in the regulation of flavonoids [47]. In *K. arborescens*, the transcription factors, such as MYB, AP2/ERF, C2C2, NAC, and WRKY, actively participated in the responses to drought, salt, and alkali stresses, exhibiting high expression levels and significant up-regulation trends across different treatment groups. The WGCNA analysis revealed that these transcription factors could play integral roles in hormone synthesis and phenylpropanoid metabolism in *K. arborescens* under the stress conditions. Specifically, the C2C2 transcription factor (TRINITY_DN13139_c0_g1) and bHLH transcription factor (TRINITY_DN30067_c0_g1) emerged as the hub genes in the yellow module, which exhibited strong correlations with the changes in the ABA content (*p* = 0.005, cor = 0.87) and the anthocyanin content (*p* = 0.05, cor = 0.7) across different treatments (Figure 6D). In the turquoise module, enriched with more genes involved in the hormone and phenylpropanoid metabolism, two MYB transcription factors (TRINITY_DN1024_c0_g1 and TRINITY_DN6353_c0_g4) interacted with the hub genes related to the stress hormones (TCH4, SNRK2, OPR, AP2/ERF, and ACS) and phenylpropanoid pathways (CSE and F3H), collectively exerting intricate regulatory functions.

The *YABBY*, *AtbHLH92*, and *AtbHLH122* genes were involved in the plant responses to the abiotic stresses through the ABA signal transduction pathway [131,132,133,134,135,136]. Under drought, salt, and particularly alkali stress, the relevant genes in the ABA pathway of *K. arborescens* were induced, with a significant increase in ABA content in the roots. Based on these findings, the YABBY and bHLH transcription factors identified in this study were speculated to regulate the *K. arborescens* responses to the stresses by modulating the ABA or other hormone synthesis and signal transduction pathways. The MYB transcription factor family was also significantly involved, with 58 differentially expressed MYBs identified under various stress conditions. The WGCNA analysis suggested that the DEGs annotated as MYB2 (TRINITY_DN1024_c0_g1) and MYB36 (TRINITY_DN6353_c0_g4) could function within the turquoise module by regulating the genes related to the stress hormones and the phenylpropanoid pathway. In poplars, *PtrMYB94* coordinated with the ABA signal transduction pathway to enhance drought tolerance [137]. In cotton, *GhMYB36* could regulate drought resistance and resistance to *Verticillium* wilt in transgenic plants by enhancing *PR1* expression [31]. Similarly, in apples, *MdMYB88* was induced by drought stress and regulated phenylalanine biosynthesis and lignin deposition, thereby enhancing the phenylpropanoid metabolism [138]. The overexpression of *MYB12* and *MYB113* stimulated the expression of downstream flavonoid biosynthetic genes such as chalcone synthase (CHS), leading to the increased production of total flavonoids; this enhanced the plant’s tolerance to salt and drought stresses [139,140]. Therefore, the MYB transcription factors may play a crucial role in the responses of *K. arborescens* to abiotic stresses. This discovery could advance our understanding of the hormone and phenylpropanoid regulatory network in this plant, providing a foundation for further exploration of the related molecular mechanisms.

## 4. Materials and Methods

### 4.1. Plant Materials and Stress Treatments

*K. arborescens* seeds were sourced from the Siziwang Base of the Inner Mongolia Academy of Agricultural & Animal Husbandry Sciences. The base is in Siziwang Banner, Ulanqab City, Inner Mongolia Autonomous Region, China (N41° 10′–43° 22′, E110° 20′–113°). After removing the villous bracts, the plump seeds were subjected to surface sterilization in 10% (*v*/*v*) H_2_O_2_ for 15 min, followed by washing with distilled water and absorption of the residual moisture using sterile filter paper. The seeds were germinated on MS medium for 10 d. Seedlings with approximately 2 cm roots were transplanted into pots filled with modified nutrient solution, with the roots immersed in the solution. The nutrient solution was refreshed every 10 d. The hydroponically grown seedlings were cultivated in a growth chamber at 28 °C/22 °C under 65% humidity and a 14-/10 h light/dark cycle (14 h light from 8 a.m. to 10 p.m.; 250–300 mmol m^−2^ s^−1^ at the canopy height) for approximately 55 d.

To determine the optimal conditions of drought, neutral salt, and alkaline salt to investigate their effects on plants under different abiotic stresses, the 55-day-old seedlings were transferred into the modified nutrient solutions containing different concentrations of PEG6000 (0%, 10%, 15%, 20%, 30%, and 40%), NaCl (0, 200, 300, 400, and 500 mM), and alkaline salt (0, 20, 50, 100, 150, 200, and 300 mM) (NaHCO_3_:Na_2_CO_3_ = 9:1 pH ≈ 9.5 [141]) for 0, 8, and 24 h, as well as 3 and 7 d. Each treatment involved ten seedlings, and the experiment was replicated three times. With the increase in treatment concentration and treatment time, the electrolytic leakage rate and malondialdehyde (MDA) content gradually increased, indicating that the increase in treatment concentration and treatment time caused the damage to the plant cell membrane to gradually increase, and the integrity and lipid composition of the cell membrane were gradually destroyed (Figures S1 and S2). The optimal stress concentrations were determined based on electrolytic leakage rates twice those of the CN group, and the optimal stress duration was selected as an MDA content twice that of the CN group [142]. Finally, 20% PEG6000 (PEG), 200 mM NaCl (NaCl), and 150 mM alkaline salt (ALK) for 1 d were selected for further experiments (Figures S1 and S2).

### 4.2. Physiological and Biochemical Index Detection

Following the stress treatments, we measured the fresh weight of the entire plant and counted the number of wilted leaves.

The specific leaf weight was determined using the leaves located in the third to fourth positions from the top to the bottom of the plant. The chlorophyll content of the leaves was measured using a SPAD-502 chlorophyll meter.

The roots, stems, and leaves of *K. arborescens* were washed and dried until they reached a constant weight for measurement of Na^+^ and K^+^ content. The samples weighing 0.1 g each were treated with 10% HNO_3_ for 8 h. The resulting supernatant from the filtered samples was analyzed using an Optima 8000 ICP-OES DV spectrometer (PerkinElmer, Inc., Shanghai, China) following the manufacturer’s instructions [143]. 

The activities of malondialdehyde (MDA), catalase (CAT), superoxide dismutase (SOD), and ascorbate peroxidase (APX) were measured using assay kits from the Keming Bioengineering Institute, following the manufacturer’s instructions.

The electrolyte permeability was measured using a conductivity meter. Fresh leaves (2–3 pieces, total 1 g) near the middle node of the seedlings were selected for the measurements (three repetitions per treatment). The leaves were rinsed three times with deionized water to remove the surface electrolytes and then incubated in 10 mL of MilliQ water in the test tubes for 3 h at 25 °C. The conductivity of the bathing solution was measured with the conductivity meter and recorded as value A. The tubes were then placed in a water bath at 95 °C for 20 min, after which they were cooled to room temperature and the conductivity of the bathing solution was measured again and recorded as value B. The electrolyte permeability for each sample was expressed as the percentage leakage: [(value A/value B) × 100]. 

The ABA content in *K. arborescens* roots and leaves was analyzed using high-performance liquid chromatography (HPLC; ACCHROM S3000). The total flavonoid and anthocyanin contents in *K. arborescens* were determined following the method described by Zhang et al. [143]. The lignin content was measured using detection assay kits from the Keming Bioengineering Institute following the manufacturer’s instructions.

### 4.3. RNA-Seq Analysis

The roots, stems, and leaves of the seedlings subjected to the 20% PEG6000, 200 mM neutral salt, and 150 mM alkaline salt stresses were collected for the RNA-Seq analysis, with three biological replicates for each treatment. All the samples were sent to Shanghai Majorbio Bio-Pharm Biotechnology Co., Ltd. (Shanghai, China) for RNA-Seq analysis. 

Total RNA was extracted from the tissues using a TRIzol Kit (Invitrogen, Carlsbad, CA, USA) (Plant RNA Purification Reagent). The qualified RNA was adopted to construct a cDNA library, and the sequencing libraries were generated by Shanghai Majorbio Bio-Pharm Biotechnology Co., Ltd. (Shanghai, China), according to the manufacturer’s instructions (Illumina, San Diego, CA, USA). The transcriptome was sequenced using a NovaSeq 6000 sequencer (2 × 150 bp read length). RNA purification, reverse transcription, library construction, and sequencing were performed by Shanghai Majorbio Bio-Pharm Biotechnology Co., Ltd. (Shanghai, China).

The raw reads obtained from the sequencing were subjected to quality control, and low-quality data were filtered. The clean reads after quality control were processed into fragments using the Trinity software (v2.4.0), where short fragments were extended to synthesize longer fragments (contigs). The overlapping fragments were merged to create a cohesive set of fragments. The transcript sequences within this set were identified and the longest transcript for each gene was selected as a unigene for subsequent functional analysis. 

### 4.4. Analysis of Differentially Expressed Genes

The gene expression levels were quantified as fragments per kilobase of transcript per million fragments mapped (FPKM). To identify the genes responsive to the stresses in the roots, stems, and leaves of *K. arborescens*, the DEGs were screened using DESeq2, with the criteria of |log_2_(FC)| ≥ 1, *p*-adjust < 0.05, and mean FPKM ≥ 1. The gene expression patterns were analyzed by clustering using the Fuzzy C-Means algorithm (Mfuzz). The functional enrichment analyses, including GO and KEGG pathway analyses, were performed using the online tool of Majorbio Cloud Platform (https://cloud.majorbio.com/page/tools/ accessed on 31 October 2024) [144,145].

### 4.5. Quantitative Real-Time PCR Analysis

The reliability of the transcriptome data was verified using quantitative real-time PCR (qRT-PCR). Twenty DEGs related to the plant hormone signal transduction and phenylpropanoid metabolism were selected for the qRT-PCR analysis. The primer sequences are listed in Table S11. The experiments were conducted using the TB Green^®^ Premix Ex Taq ™ II (TaKaRa, Dalian, China) (TLI RNaseH Plus) kit on the Rotor-Gene real-time PCR platform. The relative expression levels of each gene were calculated using the 2–ΔΔCt method [146,147].

### 4.6. Co-Expression Gene Network Analysis

Co-expression networks were constructed using the WGCNA (v1.72-1) package in R (v4.2.1). The analysis included all the DEGs related to the plant hormones, the phenylpropanoid metabolism, and the top ten transcription factor families. The high-quality genes were selected based on the standard of an expression level ≥ 0.5 in at least one sample. The network construction parameters included a threshold power of 20, a minimum module size of 30, and a branch merge cut height of 0.25 [148]. The networks were visualized using Cytoscape (v3.8.2), and the hub genes were identified using the CytoHubba plug-in of the Cytoscape software [149].

## 5. Conclusions

This study comprehensively analyzed the response of *K. arborescens* to drought, salt, and alkali stresses through biochemical index detection and transcriptome sequencing analysis, shedding new light on its adaptation mechanisms to desert habitats. The findings revealed that *K. arborescens* exhibited greater sensitivity to alkali stress than to drought and salt stresses, particularly in the root responses. Its robust stress resistance was attributed to the complex regulatory pathways. The hormone synthesis and signal transduction pathways, such as ABA and ethylene, along with the general phenylpropanoid pathway in the phenylpropanoid metabolism, the flavonoid synthesis, and the lignin biosynthesis pathways, were all pivotal under the abiotic stresses. Transcription factors such as MYB, bHLH, AP2/ERF, NAC, and WRKY played crucial roles in these stress responses. WGCNA highlighted two key co-expression modules closely associated with hormone synthesis, signal transduction, and phenylpropanoid metabolism. The hub gene analysis demonstrated the significant contributions of the bHLH and MYB transcription factors within these modules. These insights could advance investigations into the stress resistance mechanisms and genes associated with drought, salt, and alkali tolerance, enhance the understanding of desert plant resilience, and support the theoretical frameworks for ecological restoration efforts in desert environments.

## Data Availability

The RNA-seq datasets reported in our work have been submitted to the NCBI with the accession number of PRJNA1142404.

## Supplementary Materials

The following supporting information can be downloaded at: https://www.mdpi.com/article/10.3390/ijms252211891/s1.
