# Supplementary figures and images for: Transcriptional Profiling Analysis Providing Insights into the Harsh Environments Tolerance Mechanisms of Krascheninnikovia arborescens
Source: Int J Mol Sci. 2024 Nov 5;25(22):11891. doi: 10.3390/ijms252211891 (PMC11594238; doi:10.3390/ijms252211891)

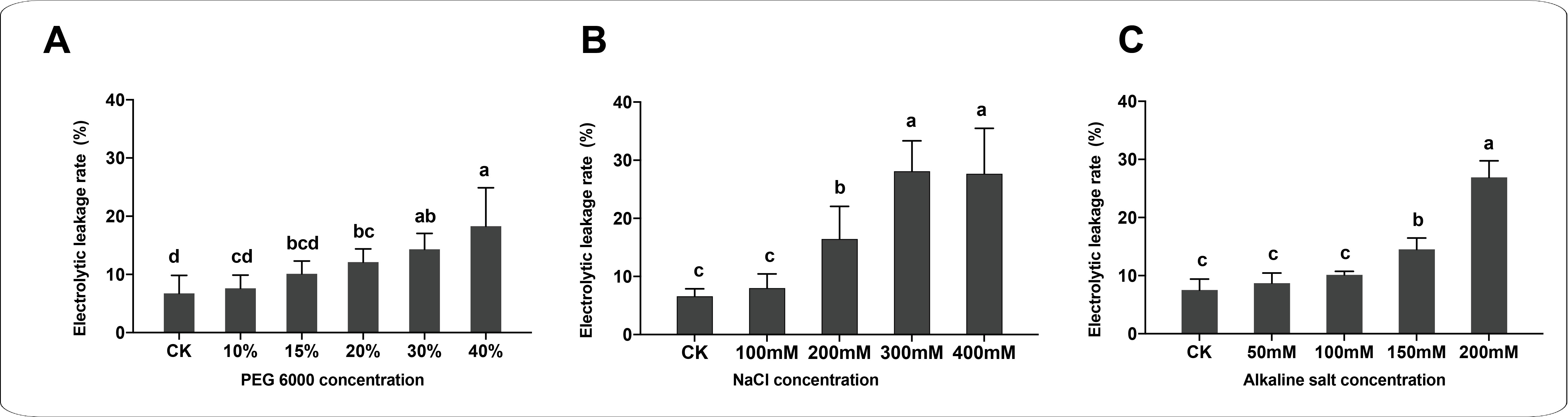

Supplement: Supplementary file 1 [file ijms-25-11891-s001.zip › IJMS_Supplementary_final/Figure S1.jpg]

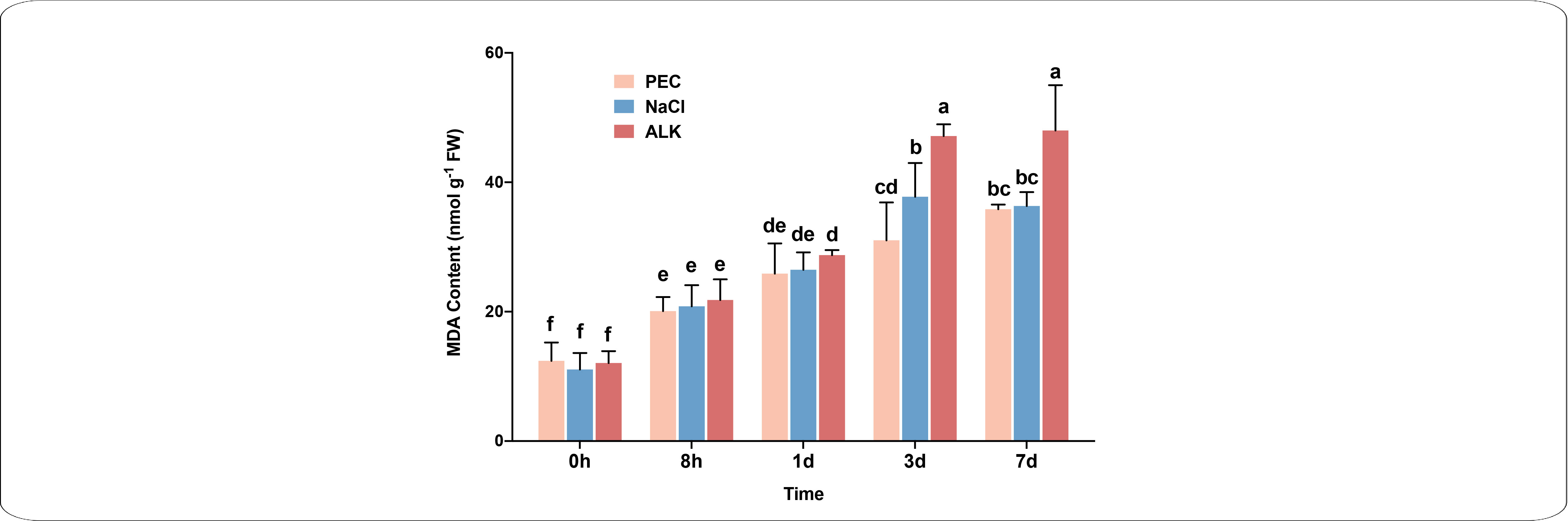

Supplement: Supplementary file 1 [file ijms-25-11891-s001.zip › IJMS_Supplementary_final/Figure S2.jpg]

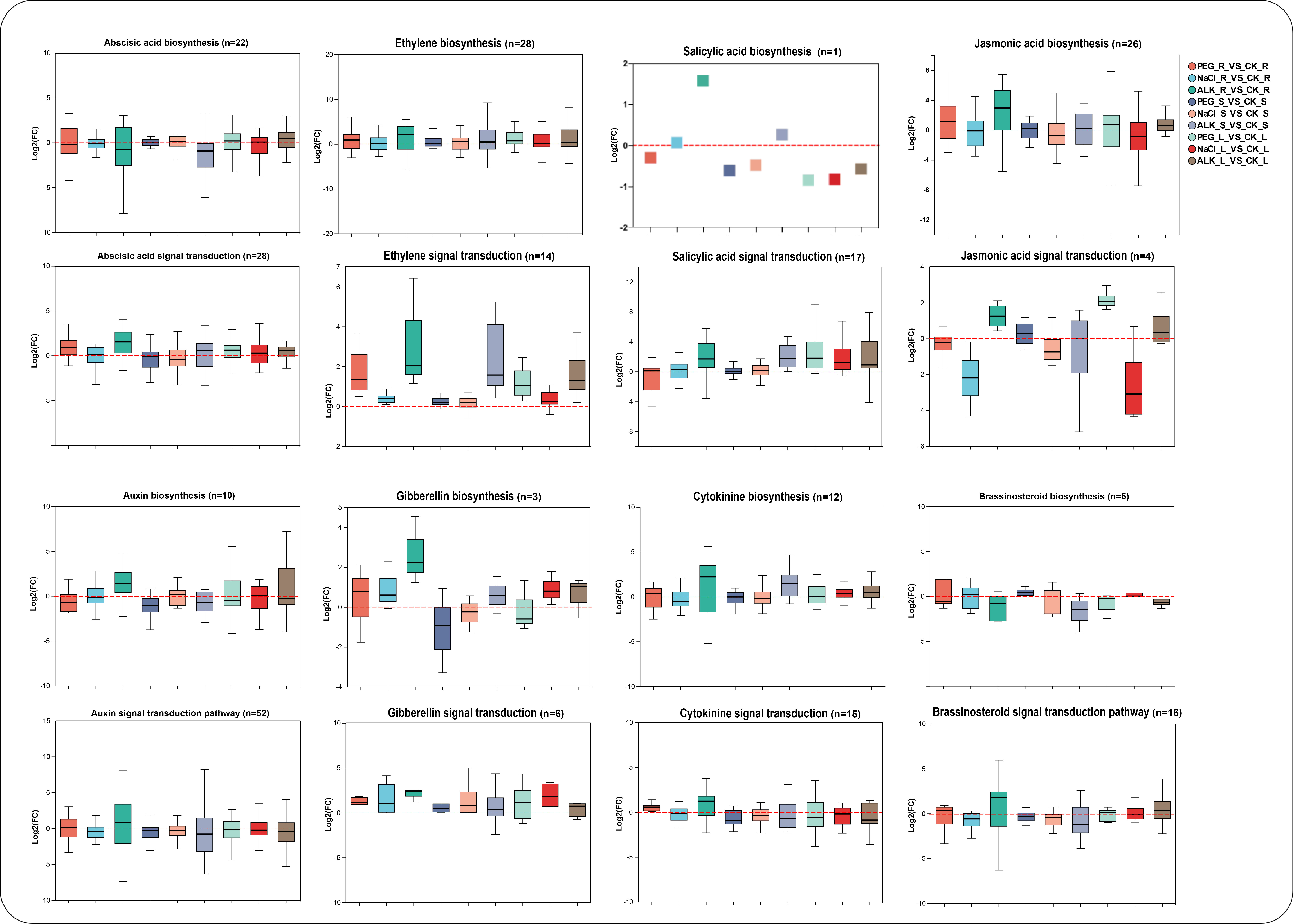

Supplement: Supplementary file 1 [file ijms-25-11891-s001.zip › IJMS_Supplementary_final/Figure S3.jpg]

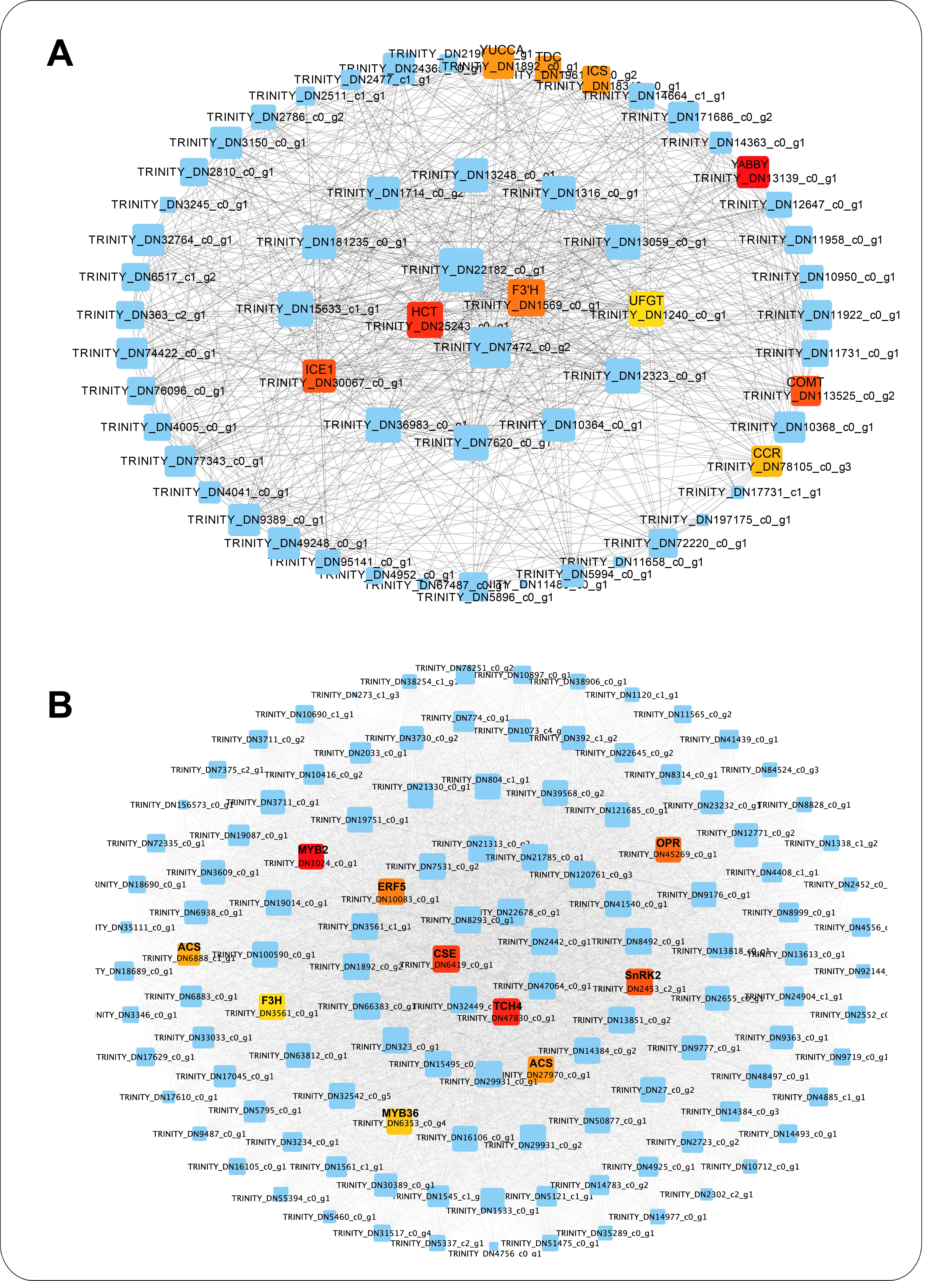

Supplement: Supplementary file 1 [file ijms-25-11891-s001.zip › IJMS_Supplementary_final/Figure S5.jpg]
